# Short-term perfluorocarbon liquid tamponade in choroidal melanoma endoresection

**DOI:** 10.1186/s40942-022-00395-z

**Published:** 2022-07-06

**Authors:** Hany S. Hamza, Ayman G. Elnahry

**Affiliations:** Department of Ophthalmology, Faculty of Medicine, Cairo University, Kasr Alainy Street, Cairo, 11956 Egypt

**Keywords:** Choroidal melanoma, Brachytherapy, Endoresection, Vitrectomy, Perfluorocarbon liquid

## Abstract

**Purpose:**

To report the use of short-term perfluorocarbon liquid (ST-PFCL) tamponade following choroidal melanoma endoresection.

**Methods:**

Patients with medium to large choroidal melanomas not amenable to primary Ruthenium-106 brachytherapy underwent choroidal melanoma endoresection following gamma knife radiosurgery. During surgery, a complete vitrectomy was performed followed by PFCL injection, then a retinectomy with endoresection of the melanoma and underlying choroid was done. Complete PFCL filling was then achieved, and laser barrage surrounding the retinectomy was done. A Ruthenium-106 plaque was then inserted. Postoperatively, supine positioning was maintained for three days followed by plaque and PFCL removal with silicone oil injection. Several months later, silicone oil was removed.

**Results:**

Four eyes of 4 patients underwent endoresection with ST-PFCL tamponade. Mean height of tumor was 8.6 ± 0.85 mm, while mean maximal basal diameter was 11.5 ± 1.1 mm. Mean preoperative logMAR best corrected visual acuity (BCVA) was 1.76 ± 0.18. All patients underwent preoperative gamma knife radiosurgery and postoperative brachytherapy. There were no major intraoperative or postoperative complications. All patients underwent silicone oil injection with PFCL/plaque removal after 3 days, while silicone oil was removed after 4 ± 1.2 months. Mean postoperative logMAR BCVA 3 months following oil removal was 0.89 ± 0.22 (p = 0.02). Mean follow-up duration was 17 ± 2.8 months. No patient developed local tumor recurrence, distant metastases, or vitreoretinal complications by final visit.

**Conclusion:**

ST-PFCL tamponade may reduce the risk of intraoperative and postoperative complications associated with choroidal melanoma endoresection.

**Supplementary Information:**

The online version contains supplementary material available at 10.1186/s40942-022-00395-z.

## Introduction

Endoresection of choroidal melanoma involves removal of the tumor using the vitreous cutter. This technique has been advocated for large choroidal melanomas not amenable to brachytherapy, tumors where radiotherapy is likely to result in optic neuropathy or maculopathy, and following radiation treatment to prevent toxic tumor syndrome [1–4].

Choroidal melanoma endoresection can be performed solely or in combination with preoperative radiotherapy and/or adjuvant brachytherapy [1–4]. Complications of endoresection include intraoperative bleeding in most cases, postoperative retinal detachment (RD) commonly due to proliferative vitreoretinopathy (PVR), local tumor recurrence, and fatal air embolism following fluid-air exchange [1, 5].

Perfluorocarbon liquids (PFCL) are heavier-than-water liquids that are mainly used intraoperatively to reattach and stabilize the retina but have also been safely used as a short-term postoperative tamponade in various retinal pathologies [6]. Among their many unique properties is their immiscibility with blood, allowing confinement of intraocular bleeding [7].

Herein, we report the use of short-term PFCL tamponade following choroidal melanoma endoresection, discuss advantages and possible limitations of this novel technique, and report its results in 4 eyes of 4 consecutive patients.

## Methods

**Fig. 1:**
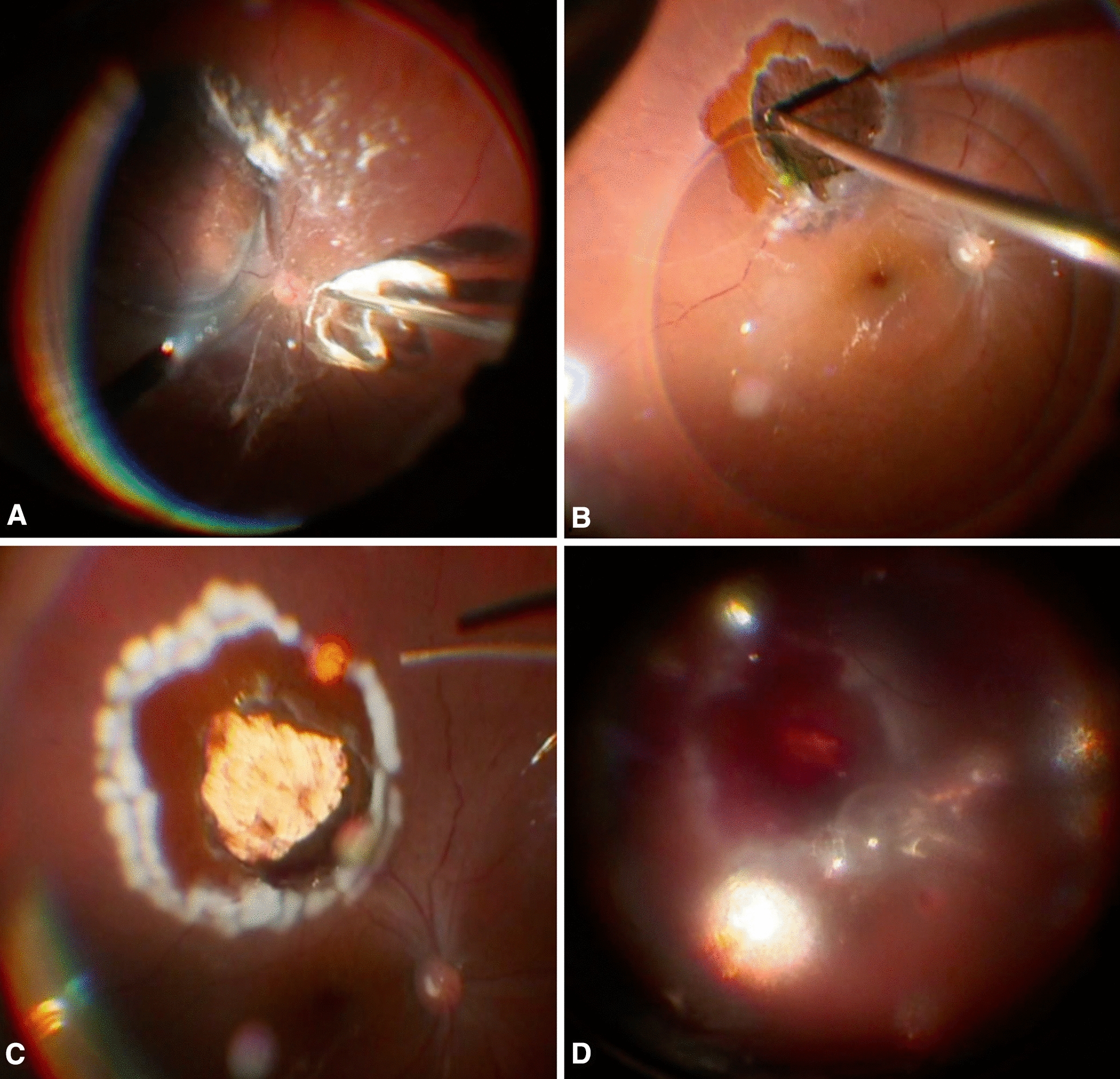
Surgical steps of the procedure. **A** Complete vitrectomy with triamcinolone-assisted posterior vitreous detachment. **B** Retinectomy and endoresection of the choroidal melanoma and underlying choroid down to the sclera in the presence of PFCL. **C** Complete PFCL fill followed by laser barraging of the retinectomy. **D** Three days later, PFCL is removed and silicone oil is injected. Note the stable blood clot overlying the psuedocoloboma

Following an average of 5 days after stereotactic gamma knife radiosurgery, patients underwent standard 3 port 23G pars plana vitrectomy (PPV) with phacoemulsification. Core vitrectomy, followed by triamcinolone-assisted posterior vitreous detachment, were first performed (Fig. 1A). This was followed by PFCL (Perfluoro-*n*-octane, FCI S.A.S, Paris, France) injection at the posterior pole to reattach and stabilize the macula. Diathermy was applied to coagulate retinal vessels, then using the vitrectomy probe, retinectomy with complete endoresection of the choroidal melanoma and underlying choroid down to the sclera was done (Fig. 1B). A cuff of seemingly normal choroid surrounding the tumor was also removed as a safety margin [1]. Hypotensive anesthesia was started around 5 min prior to endoresection and elevation of the intraocular pressure (IOP) up to 75 mmHg was done immediately prior to endoresection to ensure brief nonperfusion of the central retinal artery (CRA) and then later on as needed to decrease bleeding. Intermittent periods of CRA reperfusion, however, were also allowed during the endoresection. A complete PFCL fill was then achieved, and laser barrage surrounding the retinectomy was done (Fig. 1C). A Ruthenium-106 plaque was then inserted over the tumor bed for brachytherapy. Postoperatively, the patient was maintained in the supine position, or on his side, depending on the site of the resected tumor, so that the area of resection is most dependent under PFCL for 3 days, then the plaque was removed (after an average dose of 120 Gy was delivered to a depth of 2 mm) and air-PFCL exchange followed by silicone oil (SO) injection was done (Fig. 1D). Several months later, the SO was removed (see Additional file 1: Video S1, showing the detailed surgical steps).

## Results

**Fig. 2:**
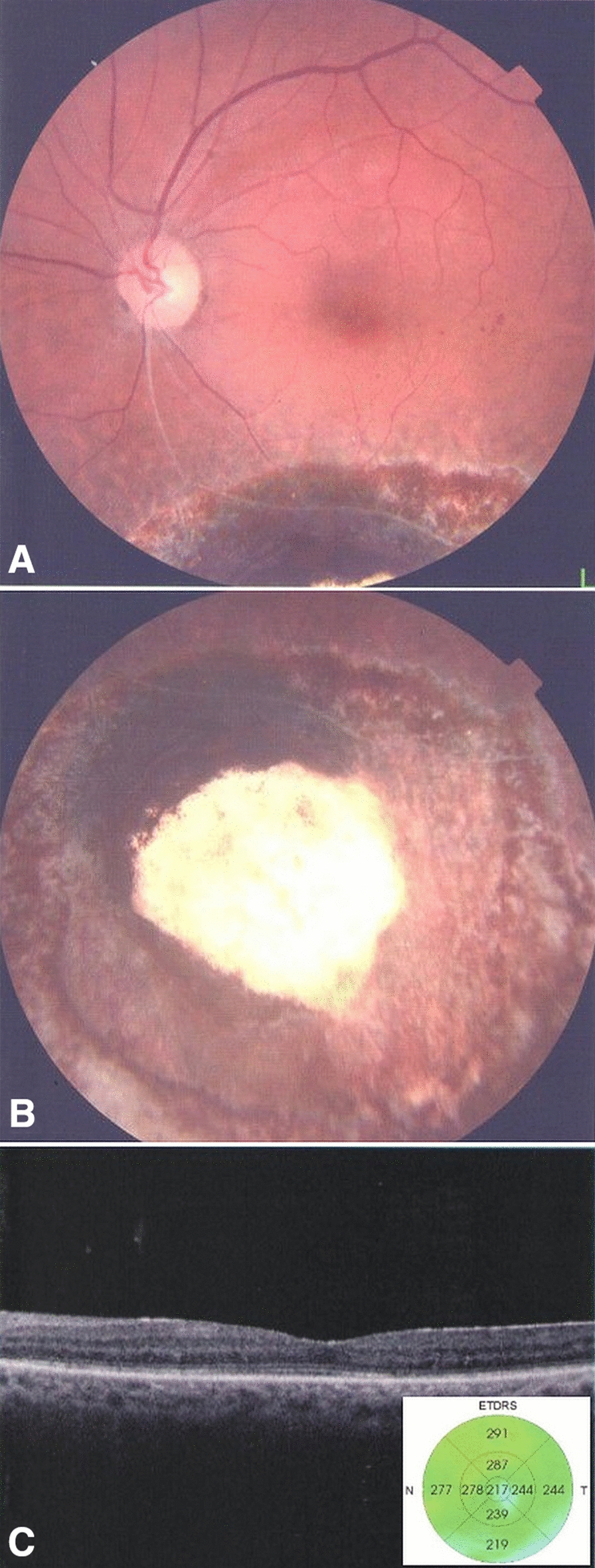
Post-operative follow-up of case 4 six months following silicone oil removal. **A** No evidence of macular traction or scarring. **B** No evidence of PVR, RD, or local tumor recurrence at the site of the pseudocoloboma. **C** Evidence of mild epiretinal membrane formation at the macula using optical coherence tomography but with no evidence of traction or retinal detachment. There is some interruption of the ellipsoid zone and external limiting membrane, but macular thickness is within normal limits (insert)

Four eyes of 4 patients with medium to large choroidal melanomas not amenable to Ruthenium-106 brachytherapy (the only isotope available in Egypt), due to insufficient radiation dose deliverable to the tumor apex, and who refused enucleation underwent choroidal melanoma endoresection with short-term PFCL tamponade. All patients signed a written informed consent to undergo the procedure. Their preoperative characteristics are summarized in Table 1. The mean ± SD age of patients was 52 ± 13.2 years. The mean height of the tumor was 8.6 ± 0.85 mm (Range: 7.8–10 mm), while the mean maximal basal diameter was 11.5 ± 1.1 mm (Range: 10–13 mm). The mean preoperative logMAR best corrected visual acuity (BCVA) was 1.76 ± 0.18. All patients underwent preoperative gamma knife radiosurgery and postoperative adjuvant brachytherapy (Ruthenium-106). There were no major intraoperative or postoperative complications in any patient. Radioactive plaque removal followed by air-PFCL exchange and silicone oil injection was done 3 days after the operation in all patients, while the SO was removed after a mean of 4 ± 1.2 months. The mean postoperative logMAR BCVA 3 months following silicone oil removal was 0.89 ± 0.22 (p = 0.02). The mean central macular thickness as measured by optical coherence tomography 6 months following oil removal was 221 ± 20 µm (Fig. 2). The mean duration of follow up was 17 ± 2.8 months. During the follow-up period, no patient developed local tumor recurrence, distant metastases, or vitreoretinal complications such as retinal traction or detachment (Fig. 2).

**Table 1:** Characteristics of patients that underwent choroidal melanoma endoresection with short-term PFCL tamponade

Case no.	Age	Sex	Pre-operative BCVA	Tumor location	Extent of RD (Clock hours)	Tumor height (mm)	Maximal basal diameter (mm)	Timing of PFCL/Plaque removal (days)	Timing of SO removal (months)	Post-operative BCVA	Central macular thickness (µm)	Duration of follow-up (months)
1	58	M	20/1200	Inferior	6	8.5	11	3	3	20/80	245	15
2	30	F	CF	Temporal	10	8.1	11.8	3	4	20/200	191	17
3	55	M	20/1200	Supero-nasal	8	10	13	3	6	20/120	232	21
4	65	M	20/600	Inferior	6	7.8	10	3	3	20/300	217	15

*BCVA* best corrected visual acuity; *PFCL* perfluorocarbon liquid; *RD* retinal detachment; *SO* silicone oil

## Discussion

Short-term PFCL tamponade has been lately used in the treatment of various challenging retinal pathologies [6, 8, 9]. Several unique properties make PFCL an especially effective tamponade. These include its higher-than-water density, high interfacial tension, and optical clarity. One property that is particularly unique to PFCL which is relevant to the currently reported technique is its immiscibility with blood. Following endoresection of choroidal melanomas, bleeding from the surrounding choroidal vessels is universal [1] and, although controllable intraoperatively in most cases, usually continues into the postoperative period. After tumor resection, attempts of direct PFCL-SO exchange may result in brief periods of hypotony that may lead to further bleeding which is then difficult to remove in the presence of silicone oil. This results in the formation of a blood clot that becomes adherent to the underlying retina which usually becomes organized with resultant PVR, retinal traction, and RD. This may be due to the inability of SO to effectively tamponade the bleeding site, owing to its lower-than-water density, and due to its increased emulsification rate in the presence of blood components [10]. Indeed, in a long term follow-up study of 71 cases of choroidal melanoma endoresection with SO tamponade in the United Kingdom, epiretinal membranes developed in 13% of eyes, while RD developed in 22% of eyes, 44% of them due to vitreoretinal scar formation. Furthermore, two cases were enucleated due to extensive PVR [1]. In another larger series of 200 cases of choroidal melanoma endoresection in Germany, 15.5% of eyes required revision PPV due to the development of complex RD after SO removal [4]. Alternatively, fluid-air exchange can be performed prior to SO injection to maintain a high IOP and decrease the risk of bleeding, however, this has been associated with fatal air embolisation [5]. These complications can therefore be mitigated by maintaining a short-term PFCL tamponade inside the eye for a few days. At the time of PFCL removal, a small but stable blood clot had formed over the tumor bed in all our cases which prevented rebleeding, and presumptively protected from systemic air embolization, during SO injection (Fig. 1D). Furthermore, any bleeding that occurred postoperatively after endoresection became confined to the retrolental space or resulted in hyphema which was easily washed away at the time of PFCL removal.

Different ocular oncologists have different approaches as regards to perioperative radiotherapy as adjuvant or neoadjuvant treatment to choroidal melanoma endoresection. At one end, some authors believe that there is little benefit from additional radiotherapy and that this leads to unnecessary radiation maculopathy/optic neuropathy [1, 11], while at the other end, others reported the concomitant use of both neoadjuvant and adjuvant radiotherapy [4]. This could be due to the different indications of endoresection for different groups. Neoadjuvant radiotherapy, however, may prevent the systemic spread of viable tumor cells during endoresection, while adjuvant radiotherapy using brachytherapy may result in a lower rate of local recurrence especially when the basal diameter of the tumor exceeds 11 mm [4, 12]. The dose of radiation delivered was also different between studies and some groups only used adjuvant radiotherapy when tumor remnants were suspected to be adherent to the scleral bed [4, 13]. The presence or absence of microscopic tumor remnants, however, can be difficult to confirm intraoperatively. For these reasons, we therefore routinely use neoadjuvant (gamma knife radiosurgery) and adjuvant (ruthenium-106 brachytherapy) radiotherapy with endoresection. Regardless of the perioperative radiotherapy approach, however, it is unlikely to influence the use of short-term PFCL tamponade which can be either removed simultaneously with the plaque, or according to the surgeon’s preference if no adjuvant brachytherapy is used.

There are a few concerns that arise with short-term PFCL tamponading. First, PFCL may be toxic to the retina and cause intraocular inflammation if used as a postoperative intraocular tamponade [14]. However, several studies have found it to be safe in the short-term, and the duration of PFCL tamponade used in our technique (3 days) is well below the duration reported in these studies [8, 9]. Furthermore, postoperative perfluoro-*n*-octane tamponade may be safer than other types of PFCL [8]. Second, PFCL may drain away through the cut ends of vortex veins following endoresection [12], which may compromise its tamponading effect [11]. However, this is rare and we did not observe it with our technique. At the time of PFCL removal, there seemed to be no appreciable decrease in the level of intraocular PFCL. Even if absorbed, PFCL is also known to be well tolerated in the systemic circulation since it was originally used clinically as a blood substitute [12].

Some surgeons will perform a peripheral retinotomy with retinal lifting in cases where the melanoma does not invade the overlying retina [13]. We prefer to remove all the retinal tissue overlying the tumor to eliminate any residual tumor cells that may have invaded the retina, especially since it is not expected that the retina will function over the created coloboma. Moreover, a large peripheral retinotomy with retinal lifting may lead to a higher incidence of macular displacement following reattachment [15], and, particularly when done inferiorly, may be associated with a higher incidence of postoperative PVR and RD. Subretinal hemorrhage may also accumulate under the intact retina. Nevertheless, we believe that short-term PFCL tamponading may work well with either procedure. In fact, short-term PFCL tamponading in these cases may have the added benefit of preventing the accumulation of submacular hemorrhage and decreasing the incidence of postoperative PVR and inferior RD.

## Conclusion

We propose that the use of a short-term PFCL tamponade following choroidal melanoma endoresection may result in a lower incidence of intraoperative and postoperative complications such as postoperative PVR and RD formation due to a possible lower risk of intraoperative and postoperative bleeding. Larger comparative studies with a longer follow-up period are needed to further evaluate the safety and efficacy of this new and promising technique.

## Supplementary Information

**Additional file 1: Video S1.** Showing surgical steps of choroidal melanoma endoresection with short-term PFCL tamponade.

## Data Availability

Data used in this study is available from corresponding author upon reasonable request.

## Abbreviations

BCVA: Best corrected visual acuity
CRA: Central retinal artery
PFCL: Perfluorocarbon liquid
PPV: Pars plana vitrectomy
PVR: Proliferative vitreoretinopathy
RD: Retinal detachment
SO: Silicon oil
